# Organic Nanocrystal Fabrication Using the Process of Resonant Second-Harmonic Generation of Light

**DOI:** 10.1021/acsomega.0c05156

**Published:** 2021-04-15

**Authors:** Andrzej Miniewicz, Michalina Ślemp, Jiri Pfleger

**Affiliations:** † Advanced Materials Engineering and Modelling Group, Faculty of Chemistry, Wroclaw University of Science and Technology, Wybrzeze Wyspianskiego 27, 50-370 Wroclaw, Poland; ‡ Department of Polymers for Electronics and Photonics, Institute of Macromolecular Chemistry, Academy of Sciences of the Czech Republic, Heyrovského nám. 2, CZ-162 06 Praha 6, Czech Republic

## Abstract

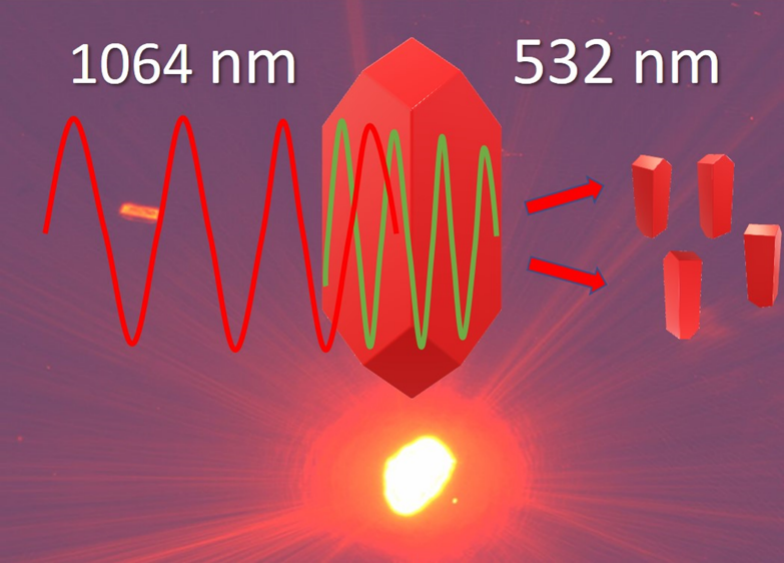

Laser ablation with the use of ultra-short laser pulses is a widely used technique for the fabrication of nanoparticles of metals, inorganic and hybrid materials. However, fabrication of fragile organic nanocrystals *via* laser ablation is rarely used due to easy photodegradation of molecules. The method employing laser irradiation of the target material is beneficial as no other chemicals are used in the production of nanoparticles, except for a given material and a solvent. In this work, we test the concept of formation of nonlinear optical (NLO) organic nanocrystals dispersion in water by irradiation of the microcrystals of the NLO material with nonabsorbed infrared nanosecond light pulses. These pulses, due to a nonlinear optical process active in a noncentrosymmetric organic crystal, such as those studied in this work, DCNP dye (3-(1,1-dicyanoethenyl)-1-phenyl-4,5-dihydro-1*H*-pyrazole), produce nanosecond pulses of second-harmonic (SH) light. Due to doubling of photon energy, they are reabsorbed in the volume of DCNP microcrystals and thermal shocks fracture them into nanometer size crystals. To the best of our knowledge, such process and its interpretation have not been described yet in the literature.

## Introduction

Organic nanoparticles, like their inorganic or hybrid organic–inorganic counterparts, may find several applications in biosensing, bioimaging, optical and nonlinear optical devices, material science and medicine.^1−7^ They are produced by various methods of chemical synthesis including precipitation,^8^ reprecipitation and solvent-vapor annealing,^9^ milling,^10^ sonication technique,^11^ laser ablation,^12−16^ size-isolation effect of dendrimers,^17^ growth in sol–gel coatings,^18^*etc.* Organic crystals are known to have large and ultrafast nonlinear optical responses and efficient fluorescence properties what makes them attractive candidates for optoelectronic devices such as optically pumped lasers,^19^ photovoltaic cells^20^ or THz generators,^21^ just to mention few examples. However, in organic crystals molecules are held together by van der Waals interactions which are much weaker than covalent bonds in inorganic crystals and, in result, they are fragile and difficult to handle, what limits their application. We focus our attention to laser-assisted processes in organic nanoparticle formation. Laser ablation is the most used technique for that purpose. In laser ablation a target material (crystalline or polycrystalline) is irradiated by pulsed laser light that results in its explosive expansion. Frequently, the target material is suspended in liquid (*e.g.* water) and laser ablation in liquids (LAL) proceeds *via* cycles of heating and cooling mediated by a liquid^39^ that is regarded as mild conditions when compared with laser ablation in vacuum. Therefore, particularly this method can be applied for organic solids to fragment them into nanoparticles. This technique was pioneered for organic material treatment already in the middle of 1990s by Masuhara group and its co-workers.^22−28^ LAL is usually performed by the impact of short laser light pulses on dispersion of larger particles in liquids, free liquid jets, or aerosols,^29−36^ leading to the formation of nanoparticle colloids. Diverse nanoparticle sizes and shapes can be obtained by controlling liquid environment, laser wavelength, pulse duration, energy fluence as well as processing time.^37−39^ Especially important is control of laser fluence and pulse duration. Short laser pulses (hundreds of fs) can easily damage bulky optical materials due to self-focusing effect or residual absorption, therefore optical damage threshold (ODT) is an important parameter determining suitability of organic crystals in nonlinear optical (NLO) and photonic applications. When light intensity level exceeds that of ODT an irreversible material damage (melting, decomposition, photodegradation, cracking, *etc.*) may occur. However, this generally unwanted effect, if well controlled, can be employed to nanoparticles fabrication *via* photofragmentation process which differs from classic laser ablation, which frequently results in amorphous nanoparticle formation. In recent work Zulina *et al.*(41) reported on laser ablation of well-known DAST NLO crystal, the obtained nanoparticles were in the form of amorphous material.^40^ Similarly Boutinguiza *et al.*(41) using long 1–3 ms pulses of Nd^+^:YAG laser have observed evaporation and rapid condensation of hydroxylapatite which formed rounded nanoparticles partially crystalline and partially amorphous. By changing laser source to cw laser he reported crystal fracturing.^41^ Another interesting possibility of single crystal fracturing has been demonstrated in the work of H–H. Fang,^42^ where femtosecond laser-induced transfer method allowed for deposition of nanocrystals on the substrate directly from a thin crystalline film.

In this work, we demonstrate, for the first time to the best of our knowledge, a unique method of organic nanocrystal fabrication from nonlinear optical chromophore microcrystals floating on the water surface by their irradiation with infrared nanosecond laser pulses. In typical laser ablation experiments, the wavelength of light is chosen to be absorbed by a material that frequently induces surface melting and surface photodegradation. In our work, we chose a laser wavelength of 1064 nm that is not absorbed by the target, but due to the second-order NLO process of second-harmonic generation (SHG) at 532 nm, the absorption of light appears as well. This mechanism guarantees very mild conditions of laser interaction with microcrystals (avoiding creation of plasma) mostly because the conversion efficiency for SHG is small and because light absorption takes place inside the crystallites and not at their front surfaces as is the usual case.

We postulate that, in this particular case, the fragmentation of microcrystals in water could proceed *via* the phenomenon of SHG. Such a phenomenon can be observed only in those noncentrosymmetric crystals in which SHG is very efficient and when second-harmonic (SH) light can be effectively reabsorbed by the crystal itself. Most materials are incapable of reabsorbing the SH light when typical laser sources are used, for example, Nd^+^:YAG laser (1064 nm), just because they are transparent at 532 nm. However, the occurrence of reabsorption of the SH wave can be easily experimentally realized in any second-order nonlinear optical crystal by a proper choice of fundamental beam wavelength, for example, using a tunable optical parametric oscillator (OPO) device pumped by a solid-state pulsed laser. In that sense, the method described in this work can be regarded as a prospective one.

## Material Characterization

**Figure 1:**
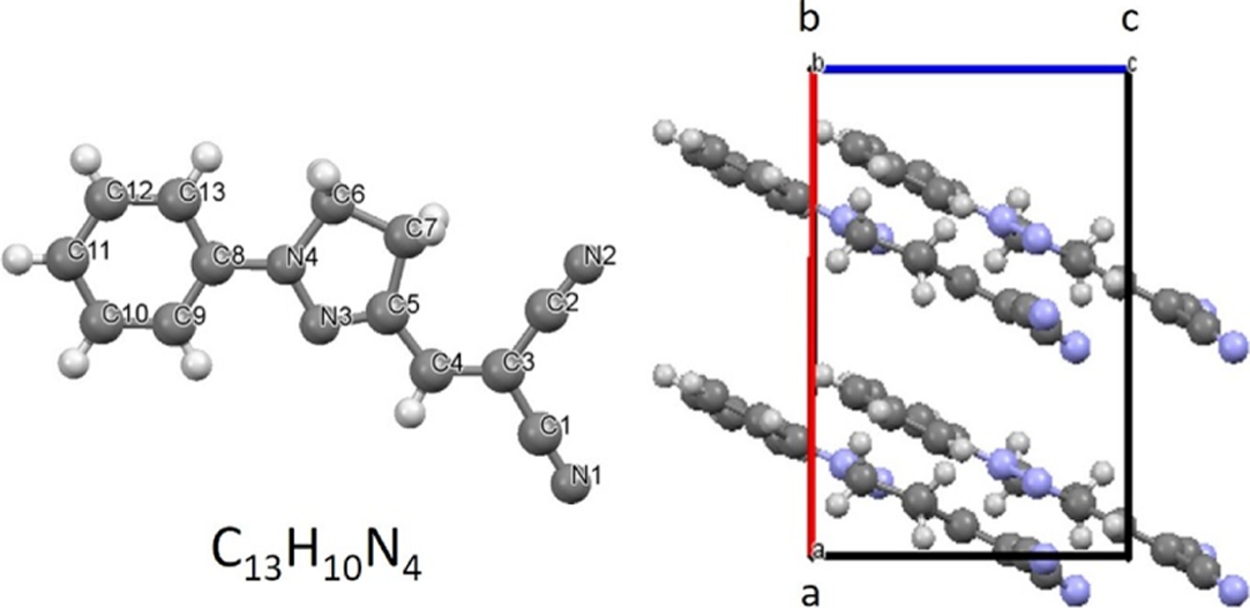
Molecular structure of push–pull NLO chromophore DCNP. Molecular packing viewed along the *b*-axis of the crystallographic unit cell of the *Cc* space group containing four molecules with its dipole moments aligned parallel to each other. Adapted with permission from Cole, J. M.; Wilson, C. C.; Howard, J. A. K.; Cruickshank, F. R. Quantitative analysis of hydrogen bonding and atomic thermal motion in the organic non-linear optical material DCNP using X-ray and neutron diffraction. *Acta Crystallogr., Sect. B: Struct. Sci*. **2000**, *56,* 1085–1093. [cif. file: Cole, J. M.; Wilson, C. C.; Howard, J. A. K.; Cruickshank, F. R. CCDC 156679: Experimental Crystal Structure Determination, 2001, DOI: 10.5517/cc5815m, Copyright 2001 IUCr Journals].

For the purpose of this work, we have chosen the noncentrosymmetric organic molecular crystal DCNP (C_13_H_10_N_4_) formed with 3-(1,1-dicyanoethenyl)-1-phenyl-4,5-dihydro-1*H*-pyrazole (or also named 3-(2,2-dicyanoethenyl)-1-phenyl-4,5-dihydropyrazole) molecules as its building blocks. The above-mentioned DCNP compound crystallizes in the monoclinic *Cc* space group as established in the work of Allen *et al.*(43) and later confirmed by neutron diffraction by Cole *et al.*(44) Then, the crystal is able to show second-order nonlinear optical properties. The molecular structure of DCNP and the projection of molecular packing in the crystal unit cell viewed along the crystallographic *b*-axis are shown in Figure 1.

**Figure 2:**
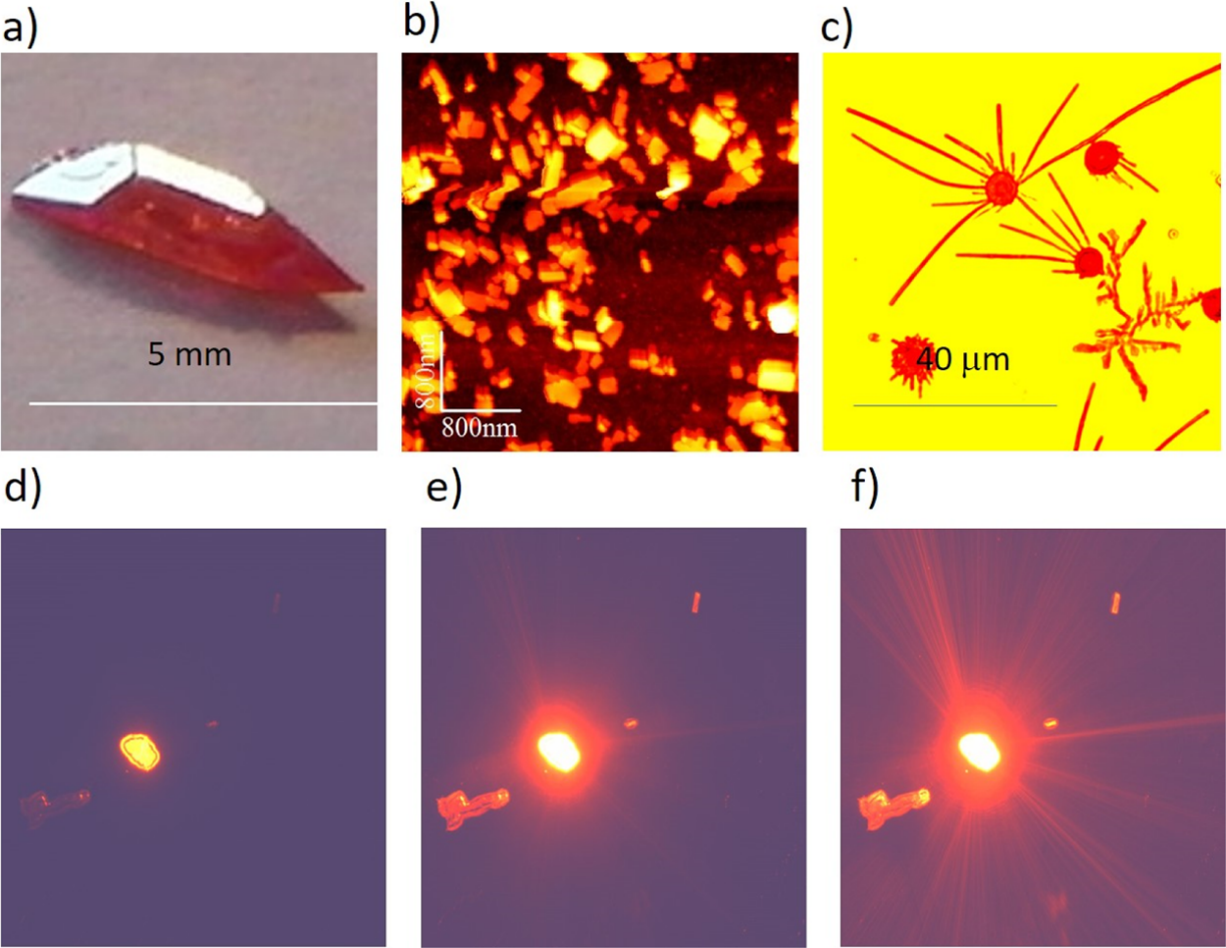
Various forms of DCNPs: (a) bulk single crystal grown from toluene solution by the slow evaporation method; (b) AFM image of DCNP nanocrystals on glass obtained from precipitation of DCNP dissolved in toluene and addition of water; (c) optical microscopic view of DCNP microcrystals confined between two glass plates and undergoing sublimation at an elevated temperature in a N_2_ atmosphere (visible growth of microbelts); (d–f) luminescence from a 40 μm irregular microcrystal of DCNP excited with a 473 nm cw laser light. With an increasing power of pumping laser from μW to mW under an optical microscope, one can observe efficient luminescence and amplified spontaneous emission showing well-directed rays in far field emerging from a crystal (cf. also refs^53,54^).

Single crystals of DCNP were obtained from a toluene solution by the slow solvent evaporation method. They are deeply red in color (the absorption edge at room temperature is located at 580 nm in the DCNP crystal^43^) and exhibit characteristic elongated shapes (cf. Figure 2a). Pure DCNP crystals melt in an oxygen-free atmosphere at 422 K. Almost parallel molecular packing in the noncentrosymmetric crystallographic unit cell with the molecular dipole moments pointing in one direction (cf. Figure 1) creates the crystal with exceptionally large linear electro-optic response (the Pockels effect). The diagonal Pockels tensor component *r*_333_ of DCNP is equal to 8.7 × 10^–11^ mV^–1^ as measured at 632.8 nm by Black *et al.*(45) This crystal possesses other very interesting optical and spectroscopic properties. As reported by Allen *et al.*,^43^ DCNP exhibits an exceptional nonlinear optical potential, giving the powder optical SHG signal approximately 100 times larger than urea when the light with a wavelength of 1.9 μm was used. SHG is also very efficient in this material even under excitation by 1064 nm pulses of Nd^+^:YAG laser as reported by Miniewicz *et al.*,^46,47^ but in this case, it is accompanied by intense red fluorescence dependent on infrared light polarization. Morawski *et al.*(48) studying low-temperature luminescence of DCNP crystals have revealed that the origin of luminescence emission in the DCNP crystal is different from molecular emission and comes from the trapping centers formed in this crystal. In order to deeply understand the DCNP properties, first-principles quantum chemical calculations of molecular and crystal electronic and vibrational excitation energies were calculated by Makowska-Janusik *et al.*(49) Further, it has been established that nanosecond pulsed laser excitation of DCNP crystallites leads to the observation of an amplified spontaneous emission (ASE) process. ASE was observed from DCNP crystallites embedded into poly-(methyl methacrylate) (PMMA) as well as from DNA biopolymer thin films.^50,51^

In Figure 2, we show the examples of bulk DCNP crystals grown from toluene solution by the slow solvent evaporation technique (Figure 2a), nanocrystals obtained from precipitation of toluene solution by adding water (Figure 2b), and also the growth of quasi-linear crystals *via* sublimation in an ambient nitrogen (N_2_) atmosphere in a confined space limited by glass plates separated by 10 μm (Figure 2c). Quite similar structures, crystalline microbelts, though obtained differently, *via* substrate-supported rapid evaporation crystallization were observed in another NLO-active noncentrosymmetric organic crystal of DAST.^52^ In Figure 2d–f, we present the optical microscopic view of the DCNP microcrystal pumped with a cw laser light of 473 nm that shows laser-like luminescence amplified by multiple reflections at its edges. The lasing properties in DCNP microcrystals have indeed been reported in the work by Cyprych *et al.*(53) The images shown in Figure 2d–f demonstrate the potential of DCNP microcrystals becoming organic microlasers when properly pumped with an excitation light as it was experimentally and theoretically considered in the work by Bittner *et al.*(54) for the DCM dye doped with PMMA and forming flat square cavities. In the case of DCNP microcrystals, we expect the formation of effective cavities as its refractive indices are relatively high: *n*_*x*_ = 1.9 and *n*_*z*_ = 2.7 at 633 nm.^45^

## Results and Discussion

### Laser-Assisted Microcrystal Fragmentation Experiment

**Figure 3:**
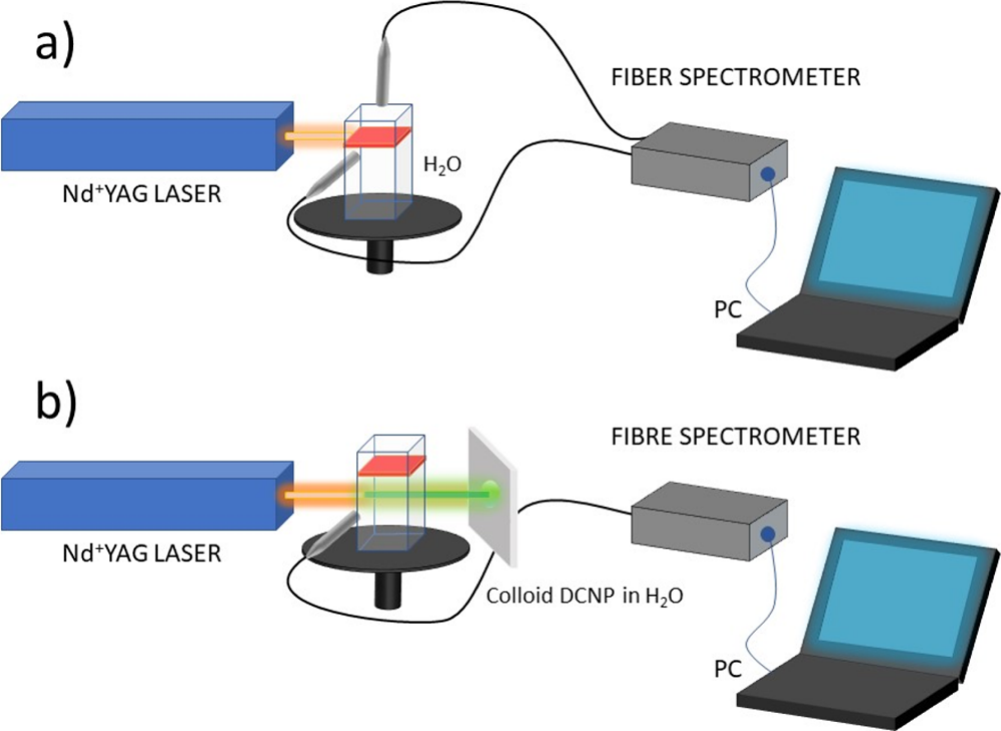
Schematic (not in scale) experimental setups showing the procedure of laser-induced photofragmentation of initial powder of DCNP microcrystals into a final colloidal solution of DCNP in water. The positions of excitation beam and optical fiber end collecting the output light are schematically shown. (a) Initial experiment (lasting 3–5 min) with the IR light hitting the DCNP layer (red color) and (b) probing the SHG signal (green color) emerging from the colloid.

The setup for the main experiment dedicated to the purpose of this work is shown in Figure 3a. We used a 1 × 1 × 3 cm^3^ optical cuvette filled with demineralized water, and a small amount of DCNP powder was added directly on the water surface. Due to the high surface tension of water, the layer of DCNP powder, thickness *ca.* 300 μm, floats on its surface. A horizontally incident non-focused laser beam of fundamental frequency ω and a 10 Hz repetition has been directed in such a way to excite this floating powder layer showing fluorescence and generation of an SH (doubled in frequency 2ω) light. For monitoring the output light, we have used two locations of the optical fiber: (i) placed directly above the powder layer and (ii) placed in the plane of the layer at an angle of 90^o^ to the laser beam (cf. Figure 3a). No lenses were used to collect the scattered light. Both SH and luminescence light that emerged from the layer were recorded, averaged, and analyzed as a function of laser light energy density (fluence). After prolonged (few minutes) illumination, the initially transparent water in the visible range becomes yellowish with almost no signs of light scattering. By moving up the cuvette by 10 mm, an incident laser beam irradiated only the water below the layer; surprisingly, the SH light was still observed from the side as well as in the forward direction on the screen (cf. Figure 3b). Appearance of SH light in solvent after the seeding experiment suggested photofragmentation of DCNP microcrystals into smaller parts and possibly forming a colloid of nanocrystals. With time, some larger nanocrystals of DCNP, which were formed by the photofragmentation, underwent the sedimentation process, but the remaining colloid of nanoparticles was stable, showing an SH signal even after few days from the photofragmentation experiment.

### Spectroscopic Results

**Figure 4:**
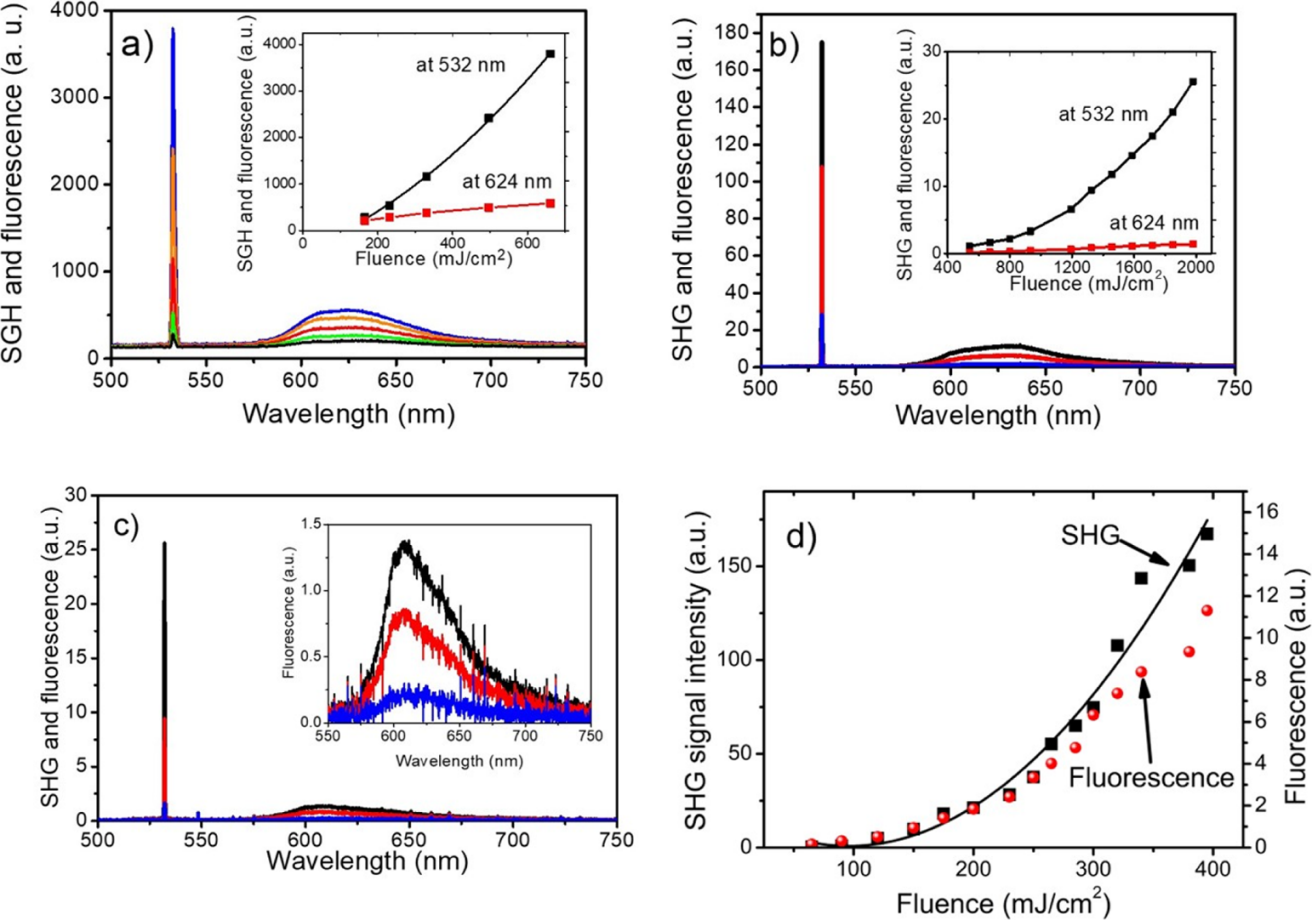
Exemplary spectra of the SHG signal (at 532.15 nm) and luminescence of DCNP powder irradiated with pulses of Nd^+^:YAG laser. (a) Spectra of DCNP powder confined between two glass plates; the inset shows their dependences on the laser fluence in the range 0.2–0.7 J cm^–2^. (b) Same type of spectra obtained from DCNP powder floating over water with the fiber end positioned over the layer; the inset shows their dependences on the laser fluence in the range 0.5–2 J cm^–2^. (c) Spectra obtained from DCNP powder floating on the water surface with the fiber end positioned in the plane of the powder layer at 90^o^ to the excitation light direction; the inset shows the shape of fluorescence bands. (d) Comparison of SHG signal and fluorescence on laser fluence for DCNP powder between glass plates showing departure of fluorescence signal from linear dependence on SH signal at about 0.3 J cm^–2^.

Before the photofragmentation process, as a reference, we have measured the intensity of the SH signal emerging from powdered DCNP microcrystals placed between two microscopic glass plates. The results of irradiation of the sample with 1064 nm, 10 ns pulses as a function of pulse energy density (0.2–0.7 J cm^–2^) are shown in Figure 4. Both narrow SHG signal at 532.15 nm and broad luminescence extending from 590 to 650 nm were observed. The sample optical response of DCNP powder is relatively strong—the red luminescence and the green SH signal can be seen with naked eyes. The joint response of NLO crystals, after single laser shot, as almost immediate SH signal and much delayed in time fluorescence light are interesting for the time resolved microscopy to study the dynamical disorder in soft materials.^55^ Next, the same DCNP powder has been delicately placed on the surface of demineralized water. The IR laser beam has been directed in the plane of the water meniscus because part of the excitation light was passing through the suspension. Due to the geometry of the sample illumination, most of the SH signal was emerging at the edge, through which light entered the cuvette accompanied by significant red luminescence. The two locations of the optical fiber end, differing by 90^o^, have been used as shown in Figure 3a. In Figure 4, we have compared the dependencies of SHG and the corresponding luminescence outputs on energy density of the input beam. The SH signals show quadratic dependence on the input laser fluence, as expected. The luminescence intensity follows the intensity rise in the SH signal, but for higher fluencies, the departures can be noticed. An example of such behavior, for DCNP confined between glass plates, is shown in Figure 4d. Two different scales were used for better visualization of this process; a significant decrease of luminescence is observed starting from the fluence above 0.3 J cm^–2^. We link this phenomenon with an increase of temperature of microcrystals due to absorption of SH light. In the work of Morawski *et al.*,^48^ the DCNP luminescence has been attributed to originate from the excitonic band, with the bottom at ∼18 115 cm^–1^, and from two main trap states having depths of ∼875 and ∼2465 cm^–1^. The shallower traps have a depth of only 0.108 eV; then, the temperature increase can lead to depopulation of these trapping states and in consequence diminish the efficiency of luminescence. The other explanation can be linked with photodegradation of the surface of DCNP crystals being in contact with oxygen at elevated temperatures; this process was observed as photodarkening of crystal surfaces during the experiments of SHG in powder confined in between glass plates.

For the DCNP powder deposited on the water surface, much higher input laser average energy densities can be used without sample damage, that is, up to 2 J cm^–2^ when compared to maximum 1.2 J cm^–2^ for powder confined between glass plates. We suppose that this difference is related to the considerably lower increase of local temperature in the former case. Water is able to absorb more heat than glass, as the specific heat of pure water (4179.6 J·kg^–1^·K^–1^) is about 5.5 times larger than that of glass (753 J·kg^–1^·K^–1^). However, also in this case, we have observed a decrease of luminescence with respect to the SH signal, but now, it occurred at much larger laser fluencies, that is, above 1.6 J cm^–2^.

We also observed the difference in luminescence band shapes for the two different geometries of light collection by the fiber end input to a spectrometer. The most pronounced one was observed for the side position of the fiber with respect to the layer of DCNP. In this case (see Figure 4c), the long-wavelength band (at 630 nm) is attenuated with respect to that at 610 nm. Using a setup shown in Figure 3b in which laser beam was centered below the DCNP layer, we could probe the water colloid phase with IR laser pulses. Unexpectedly, we observed SHG process which appeared as a weak green light scattered in the liquid bulk and as a green spot at the screen.

**Figure 5:**
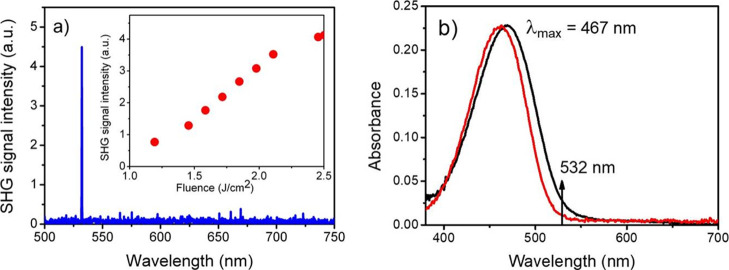
(a) Spectrum of response of water colloid with dispersed nanocrystals of DCNP to excitation by 1064 nm, 10 ns pulses of laser light; the inset shows the intensity of the SHG signal as a function of laser fluence. (b) Absorption spectra of the water colloid with dispersed DCNP particles (black line) and molecular solution of DCNP in THF (red line, DCNP absorbance in the THF spectrum was normalized with respect to the colloid one).

By positioning the fiber end aside the path of the excitation beam in the colloid, we were able to register the spectrum of scattered light, as shown in Figure 5a. The much weaker, due to lower density of crystallites, but well-measurable SH signal in this case is almost not accompanied by fluorescence. In this experiment, we could apply larger laser fluence up to 2.4 J cm^–2^ without observing any laser damage effects, but the quadratic dependence on laser fluence in this case is almost lost. In Figure 5b, we present the absorption spectrum of the supernatant after laser irradiation process which contained dispersed DCNP crystallites with probably sub-micrometer sizes. Repeating the measurement of the absorption spectrum after few days from a photofabrication experiment, we obtained a similar result concluding that no important sedimentation process had occurred during that period. A maximum of absorption spectrum of the colloid is only few nanometers bathochromically shifted with respect to the molecularly dissolved DCNP in THF, and both spectra have quite similar shapes. From this, we can conclude that the nanoparticles are formed from the same molecules as a precursor compound and no other compounds or their fragments have been created in the process. The shift of colloid absorbance when compared with the position of the absorption edge in bulk crystals (580 nm) indicates that the SH light at 532 nm is much less absorbed (cf. Figure 5b), so the efficiency of fluorescence dramatically decreases. The reason for this is that with the reduction in size, the density of excitonic trap states diminishes, and then absorption is closer to the molecular one. When DCNP crystals approach a hundred nanometer size, the surface-to-bulk ratio is considerably increasing, and an additional mechanism of luminescence quenching due to surface states becomes important. Still, another possibility is the luminescence quenching due to the interaction of nanocrystal surface with water molecules.

### Characterization of DCNP Crystallites

**Figure 6:**
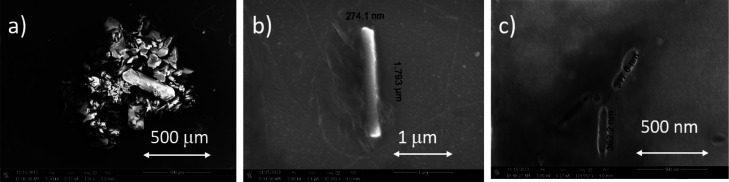
(a) SEM image of the raw material (DCNP microcrystals) used for colloid fabrication (5 kV, magnification 100×). (b) SEM image of a single rod-like DCNP microcrystal of 274 nm × 1.793 μm size (5 kV, magnification 50,201×) taken from post-irradiated solution. (c) SEM image of two rod-like DCNP nanocrystals with lengths of 362 nm and 317 nm, respectively (5 kV, magnification 119,957×), taken from post-irradiated solution.

Prior the SHG experiment, we took an SEM image of the raw DCNP powder, as shown in Figure 6a, where the scale bar is 500 μm. One can see that there is a large dispersion of size and shape, but generally, all crystallites are in between 20 and 400 μm. In the same Figure 6b,c, we present the exemplary SEM images of nanocrystals of DCNP obtained from the colloid after the photofragmentation/ablation experiment and subsequent water evaporation from a small droplet of the colloid. Interestingly, in most cases, the shapes of DCNP nanocrystals are rod-like and are of micro/nanometer size, for example, 1.793 μm in length and 274 nm in width (cf. Figure 6b), or even much smaller with lengths of 300–400 nm (cf. Figure 6c). From these SEM images, we have drawn conclusion that laser irradiation leads to formation of DCNP crystallites that preserve their noncentrosymmetricity as evidenced by the appearance of SH light in the colloid. This is an argument toward photofragmentation rather than the laser ablation process.

**Figure 7:**
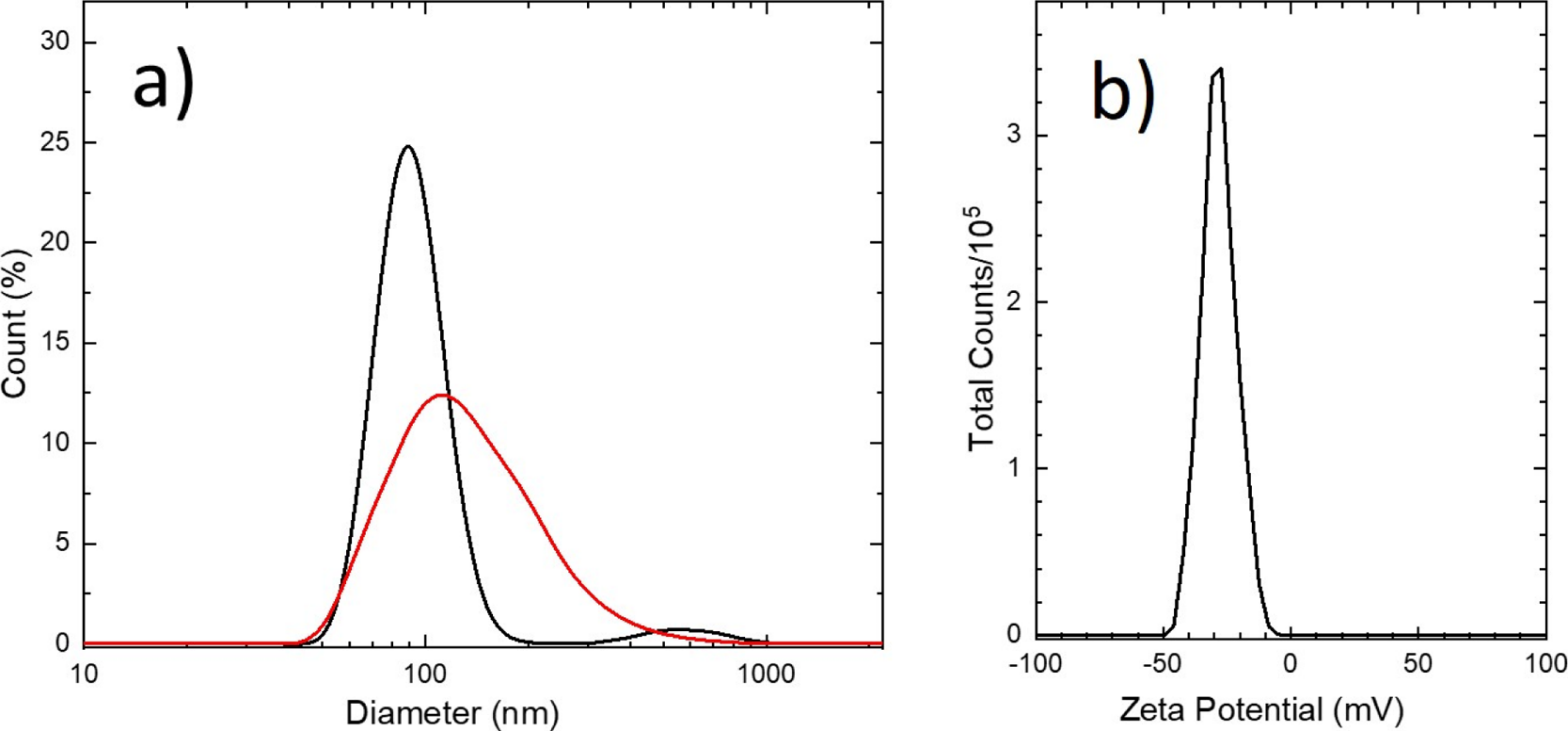
(a) Number size distribution of the colloid before (black) and after (red) filtration and (b) zeta potential distribution of the colloid before filtration. The curves were obtained by multiple curve averaging of 10 (number size distribution) and 4 (zeta potential distribution) relevant records.

In order to know the average size of the dominant in colloid crystallites, the more reliable technique must be used. Therefore, the DCNP colloid properties and the statistical distribution of particle sizes have been studied by a quasi-elastic light scattering (QELS) technique. By performing QELS experiment on nonfiltered dispersion of DCNP in water, we measured that the number size distribution was bimodal with mean values of 90 and 600 nm (see Figure 7a, black line). Next, we filtered the original colloid with a PTFE filter with a pore size of 450 nm. QELS measurements performed in this case resulted in a number size distribution of 110 nm with a shoulder at about 200 nm (cf. Figure 7a, red line). The zeta potential value was measured as ζ = −29 mV (cf. Figure 7b). The conclusion that can be drawn from the QELS experiments is that we deal with a colloid of relatively high polydispersity and average particle size slightly exceeding 100 nm. Definitely more effort must be devoted to obtain colloids of DCNP nanocrystals with lower polydispersity.

Under an optical microscope, we observed a chain-like grouping of elongated DCNP microcrystals that occurred *via* electrostatic interactions. This can be rationalized by the fact that the spontaneous polarization value *P* = 4μ/*V* in DCNP crystals is very high and amounts to 0.116 cm^–2^ as calculated on the basis of crystal structure (four axially aligned molecular dipoles with μ = 2.5 × 10^–29^ cm are confined in the unit cell of volume *V* = 1158.95 Å^3^). Such a grouping can influence the QELS experiment results producing apparently larger size distribution.

### Model of SHG-Induced Fragmentation of NLO Crystals

**Figure 8:**
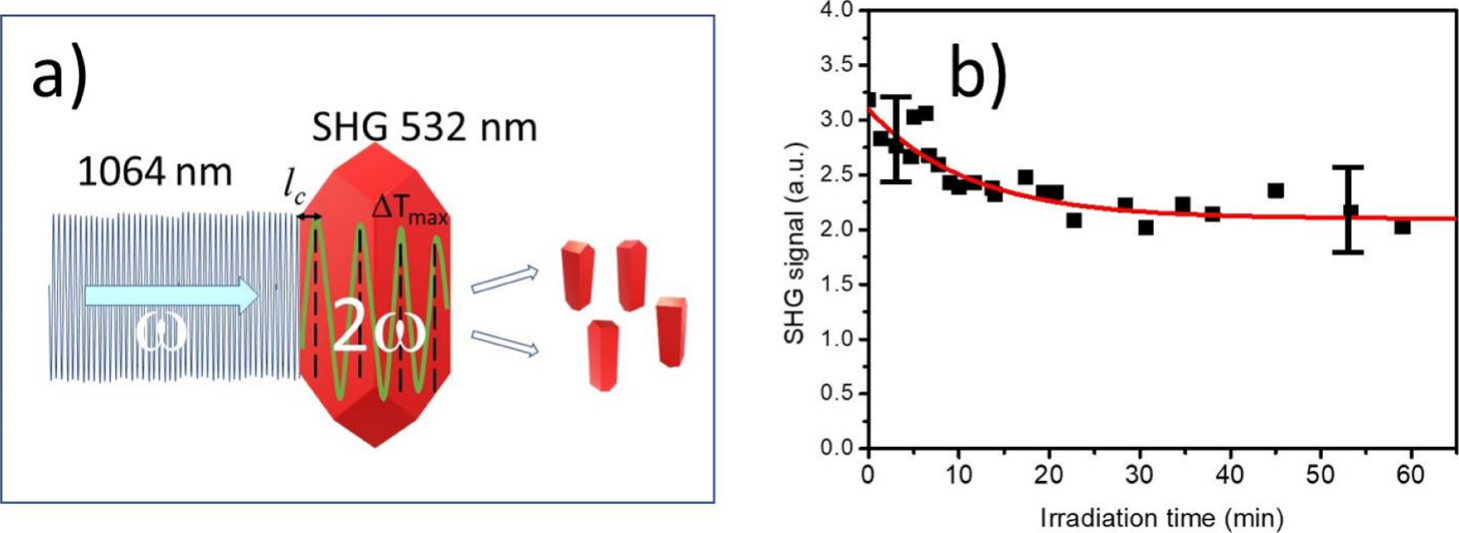
(a) Scheme of the postulated mechanism of infrared-radiation-induced photofragmentation of DCNP microcrystals *via* SHG light absorption-induced thermal stresses in the crystal bulk. (b) Dependence of SH signal intensity on time for the DCNP colloid in water under conditions of permanent irradiance with Nd^+^:YAG laser pulses of 2.46 J cm^–2^ fluence. The SH signal decay was approximated with the exponential decay function with a time constant of τ = 11 min (*I*^SHG^(*t*) = 2.1 + 1.0·exp(−*t*/τ) in arbitrary units).

In this work, we used nanosecond pulses of IR light that is not absorbed by DCNP crystals.^43^ An infrared wave of frequency ω due to nonlinear optical process of second order, *P*^(2)^ (2ω) = ε_0_χ^(2)^*E*(ω)^2^, produces an SH wave of frequency 2ω. In order to be effective, this process requires a crystal with nonzero and high value of second-order susceptibility χ_*ijk*_^(2)^ (−2ω;ω,ω). Such process, also known as resonance-enhanced SHG, is observed in the DCNP crystal investigated here, characterized among others with a relatively high component of susceptibility tensor χ_333_^(2)^ (−2ω;ω,ω) = 2206 pm V^–1^ at 830 nm.^43^ Part of the SH light is efficiently absorbed in the crystal bulk, leading to a suddenly modulated in-space temperature increase of crystallites. In Figure 8, we present a simplified view of the above-described process when the laser pulse of frequency ω is converted into a 2ω pulse strongly reabsorbed by the crystal. The envelope of the SH light intensity *I*_2ω_(*x*) oscillates with a propagation distance *x*, as counted from the crystal edge, exhibiting successive maxima and minima; the first maximum occurs at *l*_c_ and then at 3*l*_c_, 5*l*_c_, *and so forth* (where *l*_c_ is the coherence length).

The coherence length *l*_c_ is a material parameter linked with its refractive index dispersion and laser wavelength λ_ω_, describing the magnitude of phase mismatch between the wavevectors of fundamental and SH waves |Δ*k* = 2*k*_ω_ – *k*_2ω_ | in a given direction. The coherence length *l*_c_ depends inversely on refractive index dispersion^56^
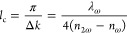
1where *n*_2ω_ and *n*_ω_ are refractive indices of an NLO crystal at frequencies ω and 2ω, respectively. Assuming *n*_ω_ = 1.8 and *n*_2ω_ = 2.1 for the DCNP crystal, one can estimate the coherence length *l*_c_ ≈ 890 nm, which is a reasonable value. In arbitrary direction of wave propagation in a microcrystal, the phase matching conditions are generally not fulfilled, that is, |Δ*k* | ≠ 0; then, the intensity of SH light oscillates with distance *x* according to the approximate equation^57^
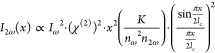
2where *K* is a constant expressing the conversion between the used system of units (cgs or SI). A small |Δ*k*| ≠ 0 value leads to a high peak intensity of *I*_2ω_(*x*) at longer distance, but a large |Δ*k*| value leads to a smaller peak intensity of *I*_2ω_(*x*) and a more rapid variation at distance *x*. On the basis of eq 2 and assuming effective absorption of SH light, we expect modulation of crystal temperature with distance *x* occurring in a relatively short time given by the laser pulse width τ_p_ = 10 ns. The thermal shock, in some crystallites, will lead to their cracking due to temperature-induced stress. We believe that the photofragmentation process most probably takes place at the coherence length *l*_c_ at which the SHG maximum is expected and consequently the highest thermal stress. Assuming single-pulse energy of 0.2 J, one gets 20 MW of power. Small-size microcrystals are able to convert only 10^–4^ of incident power into SH light, but it is sufficient to induce fracturing or bursting into smaller crystals. During prolonged irradiation with IR pulses (with repetition rate of 10 Hz) of DCNP powder, these crystals, which were fractured into sufficiently small sizes, will disperse in water to form the colloid and those that are too large will sediment. Both processes were observed during our experiments. The threshold of IR pulse intensity for nanoparticle generation depends critically on two parameters: the effective nonlinear optical coefficient for SHG process and the fragility of the crystal, both are characteristic for the studied DCNP. We noticed the coloration of the solution below the DCNP layer and the accompanying temperature rises already at fluences of the order of 1 J cm^–2^. We have found that the irradiation of the pure colloid free of any DCNP microcrystals leads to further reduction of size of colloidal particles as was evidenced by monitoring of the SH signal decay as a function of irradiation time. The example of the process is shown in Figure 8b for a laser fluence of ∼2.5 J cm^–2^. The exponential decay of the SH signal with irradiation time saturating after about 30 min evidences that post-fragmentation processing of colloidal nanocrystals is possible and that it diminishes the nanocrystal sizes, thus reducing their polydispersity.

The estimation of temperature increase due to absorption of SH light is a difficult task as it requires several assumptions and some unknown exact parameters to include in the calculations. Nevertheless, we can attempt to do that starting with an experimental parameter of energy density *E* = 2.0 J cm^–2^, which impinges on a spherical shaped crystallite of 50 μm radius. The laser energy deposited at a crystallite cross section *A* = 7.85 10^9^ nm^2^ amounts to 1.57 10^–4^ J. The volume of such a crystallite is *V* = 523 × 10^12^ nm^3^, which gives the crystallite weight *M* ≈ 666 ng using the known DCNP density^44^ ρ = 1.274 Mg m^–3^. Then, assuming that the whole incident laser energy is transformed to heat, the temperature increase in the DCNP crystallite Δ*T* ≈ 160 K has been calculated using heat capacity *C*_*p*_ = 1.5 J g^–1^ K^–1^. However, the process of SHG being dependent on phase matching conditions and on the square of light propagation length *x* which, for the discussed example, is about 100·*l*_c_ can reach about 1% conversion. Therefore, the average temperature increase of the crystallite should reach under favorable conditions at only ∼1.6 K. In the crystallite, thermal equilibrium cannot be established in 10 ns, and gradients of temperature according to the oscillatory function for SH light *I*_2ω_(*x*) given by eq 2 may introduce sufficient local stress that is able to crack the crystal. It is worth noting that similar calculations, as shown above, performed for the DCNP sphere of only 300 nm radius give ΔΤ ≈ 3200 K, and assuming in this case that SHG conversion is proportionally smaller to *x*^2^, one gets an average temperature increase due to absorption of only about 0.16 K. In our opinion, these estimations can support that the thermal mechanism due to reabsorption of SH light can be responsible for fracturing of DCNP microcrystals until nanocrystals of sizes smaller than ∼300 nm are reached.

There are other possible mechanisms to be considered, for example, a two-photon absorption process (TPA).^58^ Despite its name, this is a third-order nonlinear optical process in which four photons of the same frequency ω are involved. By definition, the probability of such a process is much smaller than the probability of the SHG process. Two-photon absorption is usually measured with laser pulses delivering intensities ∼ GWcm^–2^ and focused to observe TPEF—two-photon-excited fluorescence. In the DCNP crystal, the value of the cross section of the TPEF process was estimated to be 1.5 × 10^–50^ cm^4^ s/photon (1.5 GM).^47^ We cannot *a priori* neglect the presence of this process resulting in formation of excited states which, *via* excess energy relaxation, heat the crystallites. However, for an unfocused beam used in our experiment, the laser irradiance can be estimated from the equation *I* = *nh*ν*c*/*V* (*n* denotes the number of photons here, *c* is the light velocity, and *V* is the volume) and that for maximum average irradiance achievable from our laser *I*_max_ ≈ 0.025 GW cm^–2^ will result in the average photon density *n*/*V* ≈ 4.5 × 10^15^ (photons)/cm^3^. This photon density is far too low to observe two-photon absorption as in 1 cm^3^, we have 3.45 × 10^21^ DCNP molecules, that is, approximately 1 photon per million molecules, so the probability of meeting four photons at a single molecule is extremely low. Therefore, we decided to neglect this process at the energy fluence used in the experiment.

Another process contributing to photofragmentation to be considered is a linear absorption process of SH light produced by the neighboring molecule. The SH light is produced more efficiently when the incoming linearly polarized light of frequency ω is polarized along the optic axis of the DCNP crystal. Due to random distribution of crystallites, it happens only for a limited number of properly oriented crystals. When the SH light leaves the microcrystal, it is both scattered and absorbed by the surrounding microcrystals, and because of the 532 nm wavelength, this light is absorbed by other crystallites. The pulse width of SH is even shorter that the IR pulse, so, when absorbed, it can also induce the thermal shock leading to crystal cracking or fragmentation. The influence of this process diminishes with decreasing crystallite sizes as the SH intensity depends on the square of the distance traveled by photons inside the material (cf. eq 2). However, this process is also considered as an SHG-induced fragmentation one.

## Conclusions

Irradiation of NLO organic microcrystals with IR nanosecond pulses can lead in certain situations to their photoinduced fragmentation in an inert solvent. Such a phenomenon has been observed by us for the NLO-active noncentrosymmetric DCNP organic crystal. Prolonged irradiation of DCNP powder floating on the water surface resulted in the formation of a colloid with dispersed micro- and nanocrystals of DCNP. This has been evidenced by observation of SHG in the colloid and observation of nanocrystals by SEM and QELS techniques. We postulated the mechanism of the photofragmentation process as coming from reabsorption produced by Nd^+^:YAG laser pulses doubled in frequency light and just reabsorbed by the same crystal. Thermal stresses accompanying this process are sufficiently large to steeply fracture micrometer-sized crystals into smaller ones and finally reach nanometer sizes. The nanocrystals have been used in the SHG process as their microcrystalline precursors. The described phenomenon has not been considered before, to the best of our knowledge, to explain the fabrication of organic nanocrystals as it requires the presence of high second-order nonlinear coefficients for SHG, reabsorption of SH photons, and easy cleavage plane. However, we think that using optical parametric oscillators able to tune IR wavelengths of nanosecond pulsed laser to the value assuring that SH light will be reabsorbed, the described method can be applied to majority of organic molecular second-order NLO-active crystals. Nanocrystals showing SHG can be used in optofluidic experiments as fast markers of liquid flows in soft matter including biological cells, liquid crystals, and other liquids, provided that they are not dissolved.

## Experimental Section

SHG measurements in powder: For powdered sample excitation, we employed a non-focused linearly polarized light of angular frequency (ω) and a wavelength of λ = 1064 nm delivered by the Q-switched nanosecond neodymium-doped yttrium aluminum garnet (Nd^+^:YAG) laser (Continuum Surelite II, 10 ns pulse duration, 10 Hz repetition rate). The laser beam is linearly polarized (horizontal polarization of a 1064 nm wavelength output) with a near-field spatial profile 0.70 of a Gaussian one. The spot size of infrared radiation incident on the sample was 5 mm in diameter (measured directly from a dark spot that the laser pulse has made on the IR-sensitive paper) and the maximum pulse energy was ∼0.5 J. The pulse energies were controlled with a Q-switch, that is, time delay of Pockels cell opening. In the used geometry of SHG experiment, the entrance aperture of the fiber collected only a small portion of the SHG signal and it was positioned at an angle of 45 or 90^o^ with respect to the surface normal. Light collected by a fiber was analyzed using a spectrophotometer (Qwave, RGB Photonics) that is able to detect light in the range of 300–900 nm. We used a standard signal averaging during 300 ms over nine scans.

For the evaluation of DCNP NLO properties, we have used the Kurtz–Perry SHG powder technique.^59^ In this technique, one prepares samples in the form of powder containing a large number of randomly oriented microcrystals. We placed the DCNP powder between two microscope glass plates and sealed it. At about 532.15 nm, the single narrow line was observed that was accompanied by a fluorescent light appearing at longer wavelengths (centered at 640 nm) apparently excited by the SHG signal. The setup for the main experiment, namely, DCNP photofragmentation, dedicated to the purpose of this work is shown in Figure 3a.

Scanning electron microscopy images were taken using a JEOL JSM-6610LV SEM equipped with an Oxford Aztec Energy detector at an accelerating voltage of 5 kV. The LV mode was used at working distance (WD) of 5.2–4.0 mm; under these conditions, the resolution was ∼10 nm. After laser ablation experiment, the colloid droplet was transferred onto a carbonized substrate and allowed for water evaporation. Then, the sample was placed in the vacuum chamber and covered with a thin layer of chromium.

QELS was performed using a Zetasizer Nano ZS instrument (Malvern Instruments, U.K.). Size distribution was measured using a four-side rectangular 1 cm cuvette. All measurements were performed at a temperature of 298 K. Typically, 10–20 measurements were taken for one sample. For the recalculation of the intensity to number distributions, a reference colloid of polystyrene latex beads has been used with a refractive index of polystyrene *n* = 1.59. Water was used as a dispersant with viscosity η = 0.8872 mPa·s and refractive index *n* = 1.333 (both measured at 298 K).
